# Phylogenomic Signatures of a Lineage of Vesicular Stomatitis Indiana Virus Circulating During the 2019–2020 Epidemic in the United States

**DOI:** 10.3390/v16111803

**Published:** 2024-11-20

**Authors:** Selene Zarate, Miranda R. Bertram, Case Rodgers, Kirsten Reed, Angela Pelzel-McCluskey, Ninnet Gomez-Romero, Luis L. Rodriguez, Christie Mayo, Chad Mire, Sergei L. Kosakovsky Pond, Lauro Velazquez-Salinas

**Affiliations:** 1 Posgrado en Ciencias Genómicas, Universidad Autónoma de la Ciudad de Mexico, Ciudad de Mexico 03100, Mexico; 2 National Bio-and Agro-defense Facility, Agricultural Research Services, United States Department of Agriculture, Manhattan, KS 66506, USA; miranda.bertram@usda.gov (M.R.B.); luis.rodriguez@usda.gov (L.L.R.); chad.mire@usda.gov (C.M.); 3 Department of Microbiology, Immunology and Pathology, Colorado State University, Fort Collins, CO 80523, USA; case1prod@gmail.com (C.R.); kirsten.reed@colostate.edu (K.R.); christie.mayo@colostate.edu (C.M.); 4 United States Department of Agriculture, Animal and Plant Health Inspection Service, Veterinary Services, Fort Collins, CO 80521, USA; angela.m.pelzel-mccluskey@usda.gov; 5 Departamento de Microbiología e Inmunología, Facultad de Medicina Veterinaria y Zootecnia, Universidad Nacional Autónoma de México, Av. Universidad No. 3000 Col Copilco Universidad, Mexico City 14510, Mexico; ninnet.gomez@comunidad.unam.mx; 6 Institute for Genomics and Evolutionary Medicine, Department of Biology, Temple University, Philadelphia, PA 19122, USA

**Keywords:** vesicular stomatitis virus, evolution, positive selection, negative selection, epidemic lineages, natural selection

## Abstract

For the first time, we describe phylogenomic signatures of an epidemic lineage of vesicular stomatitis Indiana virus (VSIV). We applied multiple evolutionary analyses to a dataset of 87 full-length genome sequences representing the circulation of an epidemic VSIV lineage in the US between 2019 and 2020. Based on phylogenetic analyses, we predicted the ancestral relationship of this lineage with a specific group of isolates circulating in the endemic zone of Chiapas, Mexico. Subsequently, our findings indicate that the lineage diversified into at least four different subpopulations during its circulation in the US. We identified single nucleotide polymorphisms (SNPs) that differentiate viral subpopulations and assessed their potential relevance using comparative phylogenetic methods, highlighting the preponderance of synonymous mutations during the differentiation of these populations. Purifying selection was the main evolutionary force favoring the conservation of this epidemic phenotype, with P and G genes as the main drivers of the evolution of this lineage. Our analyses identified multiple codon sites under positive selection and the association of these sites with specific functional domains at P, M, G, and L proteins. Based on ancestral reconstruction analyses, we showed the potential relevance of some of the sites identified under positive selection to the adaptation of the epidemic lineage at the population level. Finally, using a representative group of viruses from Colorado, we established a positive correlation between genetic and geographical distances, suggesting that positive selection on specific codon positions might have favored the adaptation of different subpopulations to circulation in specific geographical settings. Collectively, our study reveals the complex dynamics that accompany the evolution of an epidemic lineage of VSIV in nature. Our analytical framework provides a model for conducting future evolutionary analyses. The ultimate goal is to support the implementation of an early warning system for vesicular stomatitis virus in the US, enabling early detection of epidemic precursors from Mexico.

## 1. Introduction

Vesicular stomatitis virus (VSV) is an arbovirus [1] that belongs to the Rhabdoviridae family and the Vesiculovirus genus [2]. VSV is a negative-sense single-stranded RNA virus. VSV genome is approximately 11 kb long and contains five genes (N, P, M, G, L), which encode five structural proteins: nucleocapsid, phosphoprotein, matrix protein, glycoprotein, and large polymerase, respectively [2]. Two main VSV serotypes have been described: vesicular stomatitis New Jersey virus (VSNJV) and vesicular stomatitis Indiana virus (VSIV), which cause numerous clinical cases in livestock in the Americas [3,4]. VSNJV is the more genetically diverse serotype, comprising six phylogenetic groups correlated with their geographical distribution [5]. In contrast, the VSIV serotype has fewer genetic clades, and the genetic differences between geographically dispersed isolates are lower than among the VSNJV isolates [6].

VSV can sporadically emerge from its endemic zones in southern Mexico and cause large epidemic outbreaks in the US [4,6,7,8]. The clinical manifestations of VSV and foot and mouth disease virus (FMDV) are indistinguishable; therefore, VSV outbreaks lead to the implementation of quarantines on infected premises until laboratory tests rule out the presence of FMDV [9]. Additionally, VSV contributes to significant economic losses associated with animal movement restrictions in the US [3,7,10,11].

Little is known about the factors associated with the emergence of epidemic VSV lineages in the US. Recent studies suggest that epidemic lineages of VSNJV represent a more virulent phenotype for vertebrate hosts than ancestral endemic lineages due to the increased ability to modulate the innate immune response [12]. Epidemic lineages of VSNJV also show an increased capacity to grow in insects in vivo [13]. Different outbreaks in the US have been consistently associated with the emergence of monophyletic lineages. However, most of these outbreaks have been investigated using partial nucleotide sequences of the P gene [4,6,7,8]. As a result, the impact of variation in other genes on disease dynamics is unknown, and comprehensive analyses of natural selective forces cannot be carried out.

The two VSV serotypes differ in their capacity to emerge and sustain epidemic outbreaks in the US. During the last 30 years, VSNJV has emerged more frequently than VSIV [4,6,7,8]. However, in 2019 and 2020, an epidemic VSIV outbreak was registered in the US following a 21-year absence of this serotype [7]. This event represents the largest US VSV epidemic outbreak in the last 40 years. During 2019, a total of 1144 premises were affected in 111 counties and eight states (Colorado, Kansas, Nebraska, New Mexico, Oklahoma, Texas, Utah, and Wyoming), with the state of Colorado recording the most instances (693 premises in 38 counties). In 2020, a new outbreak affected 326 premises in 70 counties across eight states: Arizona, Arkansas, Kansas, Missouri, Nebraska, New Mexico, Oklahoma, and Texas [7].

There is currently a significant gap in our understanding of the evolutionary relationships between endemic and epidemic Vesicular Stomatitis Virus (VSV) strains, as well as among the divergent genetic populations that emerge during epizootic events in nature. Understanding these relationships is crucial for identifying potential genetic changes that could lead to the emergence of VSV in epizootic areas and for assessing the intensity of outbreaks in these regions. Historically, the evolutionary relationships among VSV strains have been analyzed primarily through phylogenetic studies. However, there is still very little information on the role of natural selection in shaping these phylogenetic connections among genetic groups. In this context, phylogenomics serves as an extension of phylogenetics [14]. It links the evolutionary relationships among organisms with the identification of specific genomic changes influenced by genetic drift or natural selection—two recognized forces that affect the evolution of arboviruses in nature [15].

To describe the phylogenomic signatures of an epidemic lineage of VSIV in the US, we analyzed 98 full-length genome sequences from VSIV isolates collected from animals naturally infected between 2019 and 2020. To this end, we conducted a systematic evolutionary analysis using multiple high-resolution algorithms to detect natural selection. The findings from these analyses are discussed regarding the potential relevance of specific evolutionary patterns and critical genome sites that promoted this epidemic lineage’s evolution during the US outbreak. Additionally, this knowledge may help implement surveillance programs to identify potential epidemic precursors in Mexico early.

## 2. Materials and Methods

### 2.1. Viral Sequences

This study utilized a data set comprising 98 full-length genome sequences of VSIV strains from naturally infected cattle and horses. The sequences were chosen solely based on their availability in the GenBank database. For the sequences related to the 2019–2020 outbreak in the U.S., a total of 87 were included. In 2019, this set included 73 sequences collected from horses in Colorado [16], as well as three sequences from Wyoming (two from cattle and one from a horse). Additionally, there was one sequence from a horse in Texas. For 2020, the dataset included five sequences from horses in Kansas and five more from individual horses in Arizona, Missouri, Nebraska, New Mexico, and Utah. Additionally, to assess the genetic relationship of the VSIV lineage from 2019–2020 with previous VSIV strains reported in the GenBank database, we included the following sequences, representing distinct geographical clades of VSIV. From the North American clade, we utilized two sequences collected from bovines in Chiapas, Mexico (an endemic region) during 2017, two sequences recovered from infected horses during the previous VSIV outbreak in the United States (1997–1998), and the Mudd-Summers strain. From Central America, three sequences were obtained from infected bovines in Honduras (1983), El Salvador (1985), and Guatemala (1985). Finally, from the South American clade, we included sequences from infected bovines in Colombia from the years 1985, 1999, and 2001.

### 2.2. Phylogenetic Analysis

Viral genomes were aligned using CLUSTAL W [17], as implemented in the BioEdit sequencing alignment editor. Clustal W is part of a series of validated programs which are widely used in molecular biology for the multiple alignment of both nucleic acid and protein sequences and for preparing phylogenetic trees [18]. We inferred the maximum likelihood phylogeny of viral isolates in MEGA version 10.2.5 [19] using the GTR + G model as a substitution model (selected based on BIC = 62614.585) and assessed branching pattern support using 1000 bootstrap replicates. Analysis was conducted in MEGA version 10.2.5 [19]. The determination of the best substitution model for this study was conducted on MEGA. GTR + G was selected among 24 different models (Figure S1, Appendix A). Mega was used in this study based on the large repertoire of programs included in this software [19]. This software has been widely used during the last 25 years and cited in multiple studies in diverse biological fields [19]. MEGA is available as free software.

### 2.3. Ancestral Sequence Reconstruction Analysis

The Maximum Likelihood method and General Time reversible mode inferred ancestral states at specific nucleotide sites. For each node, only the most likely nucleotide sequence is shown. Initial tree(s) for the heuristic search were obtained by Neighbor-Join and BioNJ algorithms to a matrix of pairwise distances estimated using the Maximum Composite Likelihood approach and selecting the topology with superior log likelihood value. Analyses were conducted in MEGA version 10.2.5 [17].

### 2.4. Pairwise Distance Analysis

We computed pairwise p-distances between VSIV isolates using MEGA version 10.2.5 [17] and obtained sampling variance estimates by bootstrap (1000 replicates).

### 2.5. Identification of Differential Single Polymorphic Sites (SNPs)

We determined SNPs that differentiate viral populations with the Metadata-driven Comparative analysis tool (Meta-CATS) [20]. This algorithm performs a comparative analysis to identify positions in the genome that are significantly different among groups of sequences. The Meta-CATS tool was used in this study based on previous publications on the SARS-CoV-2 and Nipah viruses [21,22]. A *p*-value of 0.05 (threshold) was originally selected for this analysis. However, as shown in a previous analysis of the dengue virus using this tool [20], a Bonferroni correction analysis was carried out to reduce the likelihood of a type I error (false positives). In this sense, a conservative *p*-value > 0.0000047 was considered significant when accounting for multiple comparisons. This value was determined by dividing 0.05 by the number of sites in the alignment 11,185. Meta-CATS is free and available online.

### 2.6. Population Structure Analysis

We used the fixation index test (FST) to evaluate the extent of genetic differentiation (population structure) between different phylogenetic groups of the epidemic VSIV lineage [23]. In this context, FST values may range between 0 and 1, indicating the existence of undifferentiated (panmictic) or structured populations, respectively. This analysis was conducted using HyPhy [24], and statistical significance for FST ≠ 0 was assessed by a randomization test (1000 replicates). A *p*-value < 0.05 (threshold) was originally selected for this analysis. However, a Bonferroni correction analysis was conducted to reduce the likelihood of a type I error (false positives). In this sense, a conservative *p*-value < 0.008 was considered significant when accounting for multiple comparisons. This value was determined by dividing 0.05 by the number of comparisons carried out between genetic groups (n = 6). The HyPhy package represents a flexible and unified platform for carrying out likelihood-based analyses on multiple alignments of molecular sequence data, emphasizing studies of rates and patterns of sequence evolution [24]. HyPhy was used in this study based on previous publications on HIV, Influenza, and SARS-CoV-2 viruses [25,26,27]. HyPhy is available as free software.

### 2.7. Analysis of Molecular Variance (AMOVA)

Genetic distances were inferred using the package ape [28]; subsequently, these distances were utilized to perform AMOVA calculations using the package pegas in R [29]. AMOVA was used to estimate potential population differentiation during the evolution of the epidemic lineage. AMOVA calculates the variance between and within groups, determining the level of divergence between each other in this way. One thousand permutations were carried out to assess the statistical significance of the differences. A *p*-value < 0.05 was used as a threshold based on a previous study using the package pegas [30]. R is available as free software, providing a wide variety of statistical and graphical techniques. 

### 2.8. Evolutionary Signatures

#### 2.8.1. Identification Codons Evolving Under Natural Selection

We sought to identify specific sites in the genome evolving under natural selection using a systematic approach as previously described for SARS-CoV-2 [31]. Multiple selection detection methods, including Fixed Effects Likelihood (FEL) [32] and Mixed Effects Model of Evolution (MEME) [33], were used. These methods detect sites under diversifying and purifying selection, acting in a pervasive (FEL) or episodic (MEME) manner by inferring rates of synonymous (dS) and nonsynonymous (dN) substitutions on a per-site basis in a codon-based phylogenetic framework [34], and conduct likelihood ratio statistical tests to assess deviations from neutrality (dS = dN). FEL was used to identify codons under pervasive diversifying (dN > dS) and purifying selection (dN < dS), while MEME was used to detect both pervasive and episodic diversifying selection [35]. Analyses were conducted on HyPhy. Additionally, information about the use of MEME and FEL can be found in the following tutorial https://veg.github.io/selection-tutorial (accessed on 6 October 2024).

#### 2.8.2. Assessing the Strength of Natural Selection During the Evolution of the Epidemic Lineage

We used the RELAX test to evaluate the relative strength of natural selection during the evolution of the epidemic VSIV lineage [36]. RELAX is a general hypothesis testing approach based on a codon-based phylogenetic framework to compare the distributions of dN/dS or ω (and, thus, the selective regimes) between two non-overlapping sets of branches in a tree. The intensification/relaxation parameter K, which maps ω→ω^K, determines whether there is evidence of relaxation (0 < K<1, everything is shrunk towards ω = 1, or neutrality) or intensification (K > 1, everything is pushed further away from 1) in the test set of branches relative to the reference set. A *p*-value ≤ 0.05 was considered significant for this analysis. This value was arbitrarily selected based on a previous study describing the development of RELAX [36]. Analysis was conducted on the Datamonkey 2.0 web server [34].

#### 2.8.3. Recombination

The potential role of recombination during the evolution of the epidemic VSIV lineage was evaluated using GARD (Genetic Algorithm for Recombination Detection) [37]. This algorithm searches for the number and location of putative recombination breakpoints, which can cause potential topological incongruences in the phylogeny for different alignment parts. Differences in topology among different segments are evaluated using the posterior incongruence test (SH test) [38]. Evolutionary analyses were carried out using HyPhy v 2.5.52 [39] or later or the Datamonkey 2.0 web server [34]. A *p*-value ≤ 0.01 was considered significant for this analysis. This value was arbitrarily selected based on a previous study describing the development of GARD [37]. Analysis was conducted on the Datamonkey 2.0 web server [34].

### 2.9. Geographical Analysis

The correlation between geographical and pairwise genetic distances among different sequences obtained from Colorado was determined using the coefficient of determination (R2) analysis. For this purpose, pairwise genetic distances were calculated as described above, while hierarchical cluster analysis (HCA) was used to obtain a matrix of geographical distances among isolates. A *p*-value < 0.05 was considered significant for R2 analysis. This value was arbitrarily selected based on a previous publication [40]. Additionally, the matrix of distances was assessed using an analysis of variance (ANOVA) along with Tukey’s honest significance test to estimate the number of geographical zones in Colorado. Analyses were conducted using JMP ^®^ Pro version 16.0.0.

## 3. Results

### 3.1. The Genetic Origin of the Epidemic Lineage 2019–2020 Is Strongly Associated with VSIV Strains Circulating in Mexico’s Endemic Zones

**Figure 1:**
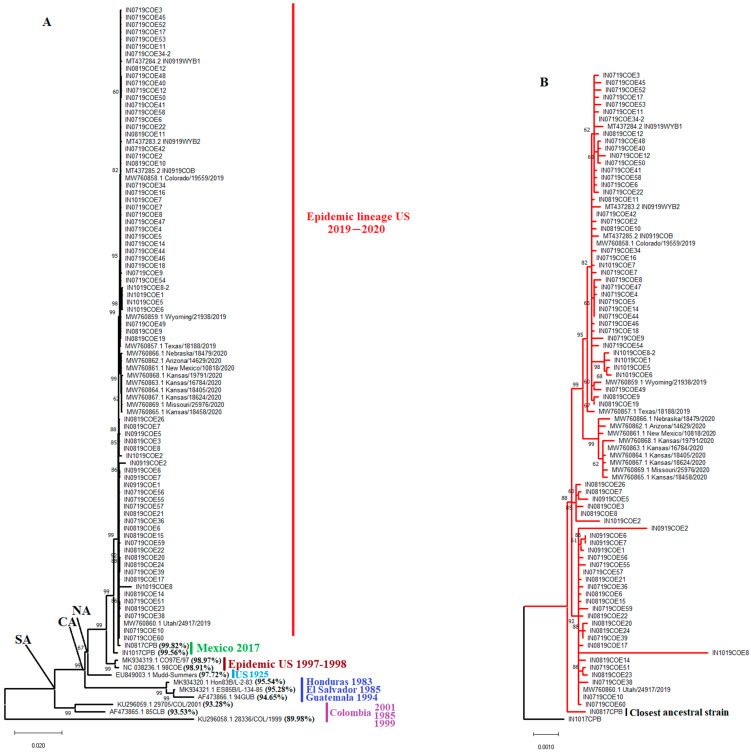
Identifying the ancestral relationship of the VSIV Epidemic lineage in the US. (**A**) a maximum likelihood tree inferred using 98 full-length genomic VSIV sequences, and the relationship between the epidemic VSIV lineage circulating in the USA during 2019–2020 and multiple earlier isolates from GenBank is shown. Branches are labeled with bootstrap support values. NA: North America, CA: Central America, SA: South America. Percentages in parentheses represent the average pairwise nucleotide identity between epidemic lineage sequences and the corresponding older isolate. (**B**) Closeup from the phylogenetic analysis showing the ancestral relationship between the epidemic lineage and isolates from Chiapas, Mexico, IN0817CPB, and IN1017CPB.

Phylogenetic analysis indicates that the 2019–2020 VSIV epidemic lineage is a conserved monophyletic lineage associated with the North American Clade (NA), with an average nucleotide identity within the lineage of 99.88% (Figure 1A,B). Based on the inferred topology, this lineage shares the most recent common ancestor and has a high degree of nucleotide similarity with the endemic Mexican VSIV isolates IN0817CPB and IN1017CPB. These isolates were recovered from cattle in the endemic region of Chiapas in 2017. Based on the topology and the lower levels of nucleotide identity predicted between the epidemic VSIV lineage 2019–2020 and the viral strains CO97E/97 and 98COE associated with the VSIV outbreak in the US in 1997–1998, we can suggest that the two epidemic events are independent (Figure 1A,B).

Interestingly, a 14 (AATTTTTTAATTTT) and 22 (AATTTTTTAATTTTAATTTT) nucleotide insertion in the intergenic noncoding region between genes G and L was also diagnostic of the epidemic lineage. The 22-nucleotide insertion was found in a minority of the isolates, including IN0819COE3, IN0819COE7, IN0819COE8, IN0819COE15, IN0819COE26, IN0919COE5, and IN1019COE2. The 14 insertion was also found in two strains from Mexico (IN0817CPB and IN1017CPB), supporting the ancestral relationship between them and the epidemic lineage. Conversely, this insertion is absent in previously epidemic VISV strains from the US (CO97E/97 and 98COE), implying two independent introductions in the US. Unexpectedly, we detected insertions in different intergenic regions associated with VSV’s highly conserved seven uracil polyadenylation signals [41]. In this context, a polyadenylation signal of eight uracil (U8) was found in the intergenic N-P region of the ancestral strain IN0817CPB. A similar insertion was also present in the Central American strain ES85B/L. Two different genotypes were found in the M-G intergenic region. On the one hand, a U8 phenotype was found in the strain IN719COE11. On the other hand, a genotype carrying nine uracil in the polyadenylation signal (U9) was found in all epidemic strains recovered during 2020, including some strains recovered during 2019 (Wyoming/219838, Colorado/19559/2019 and Texas/18188/2019). Finally, a U8 genotype was found in the G-L intergenic region of the ancestral and epidemic strains IN1017CPB and IN719COE9, respectively.

Interestingly, a comparison between the consensus nucleotide sequence of the epidemic lineage 2019–2020 and the consensus sequence obtained from the ancestral endemic strains IN0817CPB and IN1017CPB identified a highly conserved mutation (CGG(R)-CAG(Q)) at gene L codon 1784 in all viruses recovered from the epidemic lineage.

### 3.2. Epidemic VSIV 2019–2020 Lineage Diversified into Four Distinct Subpopulations in the US

**Figure 2:**
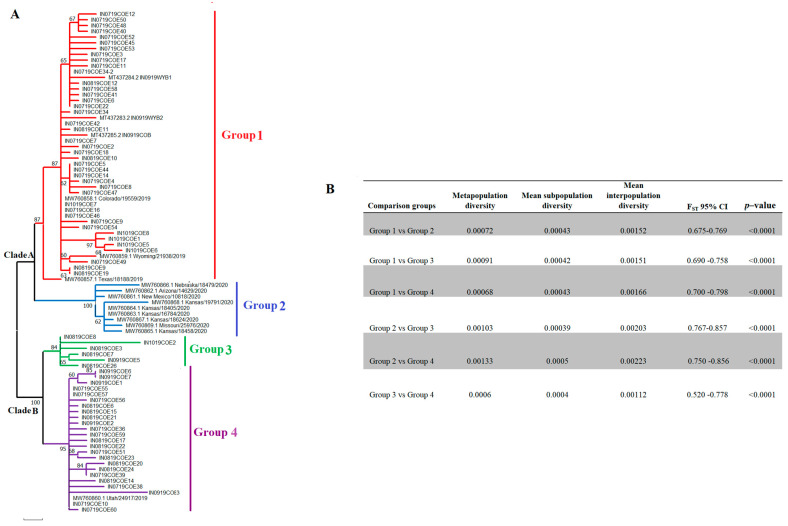
Population structure of the VSIV epidemic lineage 2019–2020 in the US. (**A**) to show the main events of diversification in the epidemic lineage during its circulation in the US, a phylogenetic analysis was conducted through maximum likelihood using a total of 87 full-length sequences representing the circulation of an epidemic VSIV lineage in the US between 2019 and 2020. (**B**) Fixation index test (FST) analysis supporting the existence of four divergent groups.

Within the epidemic VSIV lineage, 2019–2020 are two main phylogenetic clades, from which at least four distinct genetic groups can be identified (Figure 2A). The segregation into four groups is evidenced by high bootstrap values (84–100) for the corresponding internal tree branches and the results of an FST analysis (Figure 2B). Therefore, this lineage diversified into multiple subpopulations during its spread in the US. Additionally, population diversification of this epidemic lineage was confirmed for the analysis of molecular variance (AMOVA) (*p*-value 0.015).

**Figure 3:**
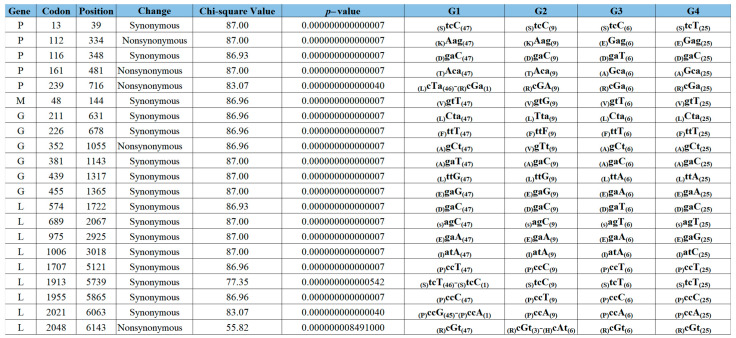
Metadata-driven comparative analysis. The SNPs associated with specific codon sites that are significantly different from the null expectation among phylogenetic groups (*p*-value of 5 × 10^−6^) were identified by the Metadata-driven comparative analysis. G1 to G4 columns represent the codon composition of different phylogenetic groups. Specific SNPs at each codon are highlighted in capital letters. The column position indicates the nucleotide position in the coding sequence at specific genes where the SNP was identified. Parentheses on the left (right) indicate the amino acid encoded and the number of sequences associated with this codon at any specific group, respectively.

Clade A gave rise to two groups, I and II. Group I comprises a total of 42 viral sequences from Colorado obtained from horses between July and September of 2019, three sequences from Wyoming, one recovered from a horse (Wyoming/21938/2019), two from bovines (IN0919WYB1, IN0919WYB2) between July and September of 2019, and one viral sequence from Texas (Texas/18188/2019), recovered from a horse in June of 2019 (Figure 3). The basal position of Texas/18188/2019 concerning the main cluster A indicates the ancestral relationship between this virus and viruses circulating in Colorado and Wyoming. Group II included nine horse isolates recovered from Arizona, Kansas, Missouri, Nebraska, and New Mexico, and it was the dominant lineage in 2020. Clade B comprises genetic groups III and IV. Group III includes a total of six horse isolates recovered from Colorado between August and September of 2019, while group IV contains viral sequences collected from horses in Colorado (*n* = 24) and Utah (*n* = 1) between July and September of 2019. Interestingly, all viral isolates showing the 22-nucleotide insertion in the G-L intergenic region were comprised in group III. No evidence of the circulation of viral lineages associated with groups I, III, and IV has been detected since 2019.

Furthermore, we identified 21 SNPs associated with the epidemic lineage’s diversification into four viral subpopulations (Figure 3). P, G, and L genes accounted for most of the SNPs found in the genome. A total of 16 out of 21 SNPs were associated with synonymous mutations, while just 5 SNPs were producing nonsynonymous changes. Interestingly, 60% of the nonsynonymous changes were located at the P gene at codons 112, 161, and 239 (Figure 3), showing the potential relevance of this gene in the diversification of this epidemic lineage. To get more insights into the significance of these SNPs in the diversification of different genetic groups, we conducted an ancestral sequence reconstruction analysis. The results were consistent with a pattern of focal evolution at specific internal nodes followed by a period of conservation preserved across the leaf branches, indicating that these SNPs survived multiple transmission events and may promote the adaptation of the epidemic lineage at the population level (Figure S2, Appendix A). Interestingly, the fixation of five SNPs located at codons P-112, P-161, G-439, G-455, and L689 were located very deep in the internal nodes promoting the divergence of the epidemic lineage in two main phylogenetic clades, suggesting the relevance of these SNPs during the early adaptative evolutionary events of this lineage (Figure S2, Appendix A).

Five SNPs at codons P-112, P-161, G-439, G-455, and L-689 were associated with the diversification of this lineage into two main clades. On the other hand, three SNPs were identified at internal nodes of G1 (P-239, G-381, and L-2021), eight at G2 (M-48, G-211, G-226, G-352, L-1707, L-1913, L-1955 and L-2048), two at G3 (P-116 and L-574), and three at G4 (P-13, L-975 and L-1006). Interestingly, some synonymous mutations at codons P-13, G-381, L-574, L-689, L-1913-, L-1955, and L-2021 were tracked in previous isolates from Central and South America origin. In the case of nonsynonymous mutations, just L-2048 was found in a lineage from South America (Figure S2, Appendix A).

There was no statistical evidence of relaxation or intensification of the strength of selection among different groups (RELAX test), suggesting that different groups evolved under evolutionary constraints indistinguishable using the available data.

### 3.3. Episodic Diversifying Selection Is a Distinctive Evolutionary Hallmark of VSIV in Nature

**Figure 4:**
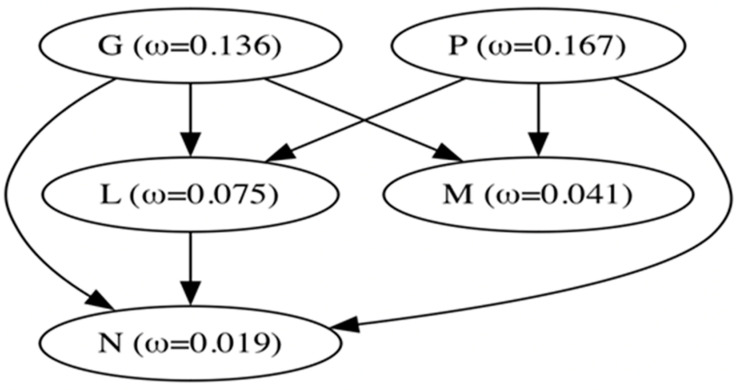
A partial ordering of VSV genes based on their average conservation (mean ω)**.** A directed arrow between gene X and Y is a statement that ω (X) > ω (Y) with statistical significance (*p* ≤ 0.05).

We used codon phylogenetic models to quantify selection pressures on viral genes, using the maximum likelihood tree inferred from full-length genomes (Figure 1). Overall, all genes were subject to purifying selection, on average (Figure 4), with all estimates significantly different from dN/dS = 1 (neutrality). Using a pairwise likelihood ratio test procedure at *p* ≤ 0.05 with a Holm–Bonferroni correction [42,43], we obtained a partial ordering of genes based on their average level of conservation, shown in Figure 4. Genes N and M were the most conserved, while genes G and P were the least conserved.

**Figure 5:**
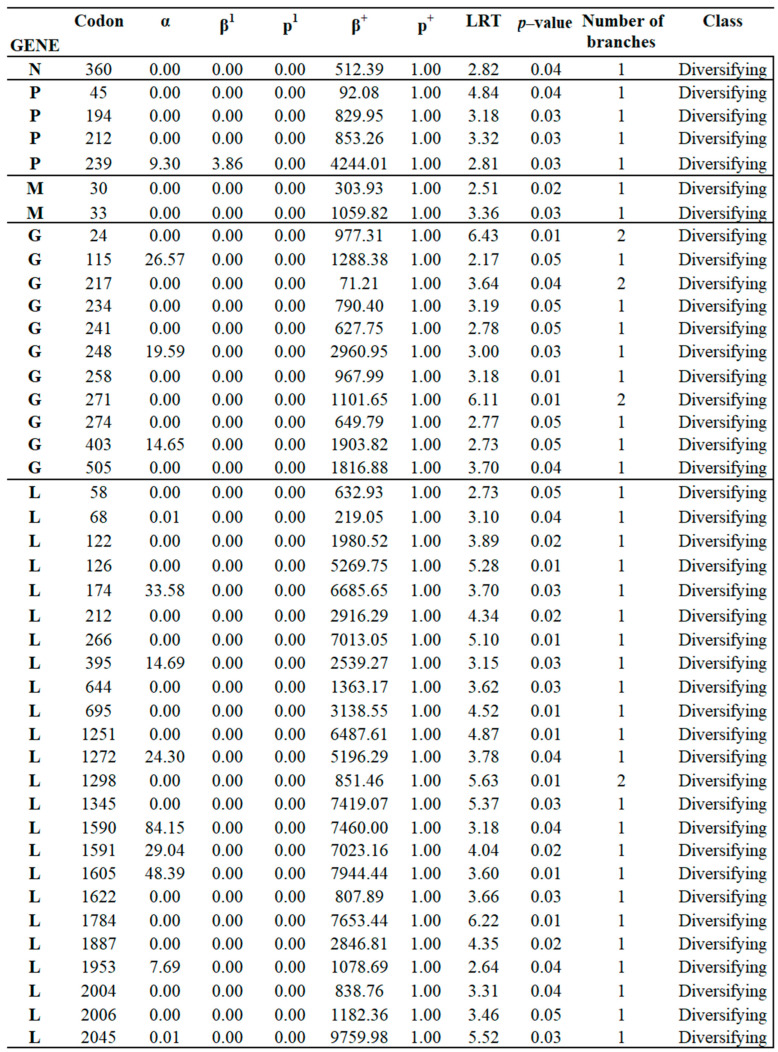
Identification of codon sites evolving under positive selection in natural populations of VSIV. The figure shows the 42 codons under positive selection identified at multiple genes VSIV by MEME analysis. α: synonymous substitution rate, β^1^:Non-synonymous substitution rate for the negative/neutral evolution component 1, p^1^: mixture distribution weight allocated to negative/neutral evolution component 1, β^+^:non-synonymous substitution rate at a site for the positive selection component, p^+^:mixture distribution weight allocated to the positive selection component, LTR: likelihood test statistics for episodic diversification, i.e., p^+^ > 0, *p*-value: asymptotic p-value for episodic diversification, i.e., p^+^ > 0, # branches: the (very approximate and rough) estimate of how many branches have been under selection at this site, i.e., had an empirical Bayes factor of 100 or more for the β^+^ rate, q: and class: selection kind.

Because of relatively low levels of diversity (tree lengths measured subs/site), we used versions of the FEL and MEME site-level detection methods, which are more suitable for the small sample, low diversity setting; they utilize parametric bootstrap with 100 replicates to assess significance [44]. Similar to the rankings based on comparing mean dN/dS estimates, genes N and M were the most conserved (largest fractions of negatively selected sites, smallest fractions of positively selected sites), while genes G and P were the least conserved. Overall, the more sensitive versions of FEL and MEME found evidence of purifying selection (at *p* ≤ 0.05) on 565 sites and of positive selection on 42 sites across the entire genome (Table 1). Specific sites under positive and purifying selection are shown in Figure 5 (Appendix B) and Figure S3 (Appendix A), respectively.

No breakpoints were detected by the GARD analysis, indicating that recombination did not play a significant role during the evolution of this sample of VISV genomes.

### 3.4. The Evolution of the Epidemic VSIV Lineage Is Constrained by the Functionality of Its Proteins

**Figure 6:**
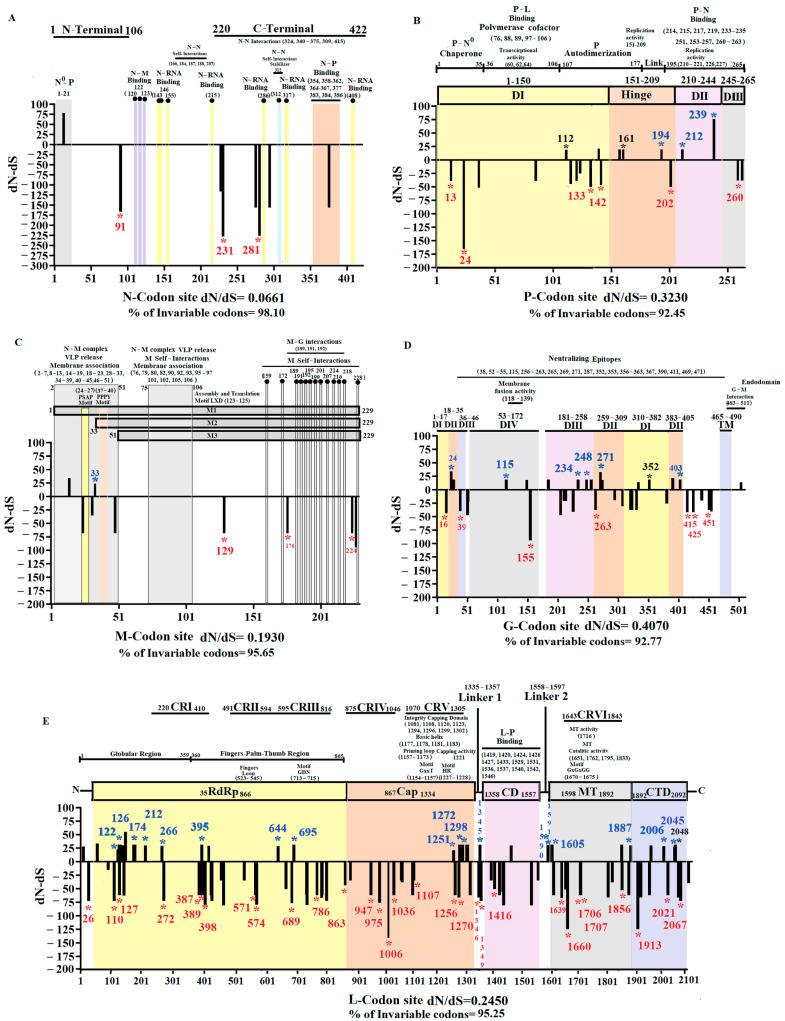
Functional gene evolutionary dynamics of the epidemic VSIV lineage. Graphics represent the dN-dS ratios for specific codon sites at (**A**) Gene N (nucleoprotein), (**B**) Gene P (Phosphoprotein), (**C**) Gene M (Matrix protein), (**D**) Gene G (Glycoprotein), and (**E**) Gene L (Large polymerase). Analyses were conducted using SLAC. Codon sites under positive and purifying selection (identified by MEME and FEL) are highlighted at specific black bars with green and red asterisks, respectively. Similarly, the specific gene location of these codons is indicated by blue and red numbers. Bars highlighted by black asterisks and numbers indicate codon sites identified as relevant by the Metadata-driven comparative analysis but evolving under neutrality based on MEME and FEL analyses. Information about functional sites, relevant motifs, and residues encoded by multiple codon sites at different genes are also indicated. Numbers in parentheses indicate codon positions linked with key residues associated with diverse functions in the viral proteome. The information about functional sites at different viral proteins was obtained from the following publications: Nucleoprotein [45,46,51,52,53], Phosphoprotein [54,55,56,57,58,59,60], Matrix protein [47,49,61,62,63], Glycoprotein [50,64,65,66,67,68,69], and Polymerase [70,71,72,73,74].

Once we determined specific codon sites at different genes under positive and purifying selection on VSIV populations in nature (Figure 5), we focused on understanding how these codon sites impacted the evolution of the epidemic lineage in the US. To comprehend if functional protein constraints might influence the location of codons under selection, we conducted an extensive literature review about previously reported functional sites at different VSIV proteins (Figure 6).

Based on dN/dS ratios and the percentage of invariable codon sites, N was the most conserved gene during the evolution of the epidemic lineage (Figure 6A). No codons were detected under positive selection in N. Overall, we observed high conservation in codons encoding critical residues associated with viral RNA interactions, N–M or N–P protein interaction, and N-self interactions (Figure 6A). A single nonsynonymous mutation affecting a unique isolate from group 4 (IN0819COE20) at codon 13 (GTC**_(V)_**−ATC**_(I)_**) was tracked on this gene, located at the N- terminal site of N protein specifically in the functional site N0-P, associated with a binding site that prevents nascent N molecules from self-assembling and from binding to cellular RNAs [45]. Three codons were found under purifying selection, none associated with a residue implied in a functional site.

The P gene appeared as the second most divergent gene in the epidemic lineage (Figure 6B). However, nonsynonymous mutations accumulated at specific functional regions, and high conservation was seen in residues associated with P–N0 chaperone region, P–L interactions (polymerase cofactor, transcriptional activity), and P–N binding. Three nonsynonymous mutations (P-112, P-161, P-239) previously predicted by our Metadata-driven comparative analysis (potentially promoting the adaptation of this lineage at the population level) were tracked in this gene. Mutations at codons P-112 and P-161, located in domain I and hinge region (both parts of the autodimerization region of the P protein) (Figure 6B), were categorized as neutral changes by MEME and FEL analyses. Conversely, codon P-239 (Figure 3) was under positive selection (Figure 5). The residue encoded by this codon is in domain II, close to codons P-233–235, encoding residues implicated in the binding between P and N proteins [46] (Figure 5 and Figure 6B).

Additionally, MEME and FEL detected codon sites P-194 (Hinge region) and 212 (domain II) under positive selection. The selection at these codon sites affected single isolates belonging to groups 2 and 1, respectively (Figure S4, Appendix A). A total of 6 codons were detected under purifying selection on this gene, preserving residues encoded by these codons at different functional sites on the P protein (Figure 6B).

The M gene showed high conservation during the evolution of the epidemic lineage in the US (Figure 6C). We noticed that sites associated with functional regions in the N–M complex, viral release, membrane association, M–G interactions, and M self-interactions displayed high overall conservation. However, two nonsynonymous mutations were predicted in this gene, affecting single isolates at two different functional sites. The first mutation at codon 14 (GGT**_(G)_**−AGT**_(s)_**) was found in the isolate IN0819COE12 (group 1) and was characterized as a neutral evolving change by MEME and FEL. Interestingly, this mutation is located in a previously predicted functional motif spanning residues 14–19 (Figure 6C), which impairs the release of viral particles from infected cells [47]. The second mutation was located at codon 33 on a single isolate of group 1 (Figure S4, Appendix A) and was predicted to be under positive selection (Figure 5). This mutation disrupts the highly conserved ATG codon, which is linked to the expression of M2, one of the three recognized forms of the M protein [48,49]. Additionally, three codons were predicted to be under purifying selection (Figure 6C).

The G gene was the most divergent in the epidemic lineage (Figure 6D), with mutations accumulated at specific locations along different functional domains (DI-DIV). High conservation was observed in the transmembrane domine (TM) and residues associated with G–M interactions. A total of six codons under positive selection were predicted on this gene (Figure 5). Three mutations at codons 24, 271, and 403 were located at DII (Trimerization domain). At codon 24, we detected mutations in single isolates from genetic groups 1 and 3. However, in each isolate, the dominant codon CAC**_(H)_** was changed to either AAC **_(N)_** or CGC**_(R)_**. Similarly, at codon 271, codon GAA**_(E)_** changed to GGA**_(G)_** or GAC**_(D)_** in single isolates from groups 1 and 4. Mutation at codon 403 was linked to a single viral isolate from group 2 (Figure S3, Appendix A). In domain III (DIII/pH domine), codons 234 and 248 were under positive selection. Mutations at these codons were found independently in single isolates from genetic groups 1 and 4. Finally, an additional site under positive selection was found in domain IV (DIV/fusion domain) at codon 115. The mutation at this codon was in a single isolate from group 1 (Figure S4, Appendix A). Interestingly, codons 115 and 271 were associated with residues recognized as neutralizing epitopes [50]. In this context, residue encoded by codon 352, located in Domain I (DI/lateral domain), was also recognized as a neutralizing epitope. This residue was previously identified through our Metadata-driven comparative analysis (linked with the emergence of G2) (Figure S1, Appendix A) and classified as a neutrally evolving site by our MEME and FEL analyses. A total of seven codons in this gene were identified under purifying selection (Figure 6D).

The gene L was the third most conserved gene during the evolution of the epidemic lineage (Figure 6E). A high level of conservation was observed in the conserved regions II, IV, and VI (CRII, CRIV, and CRVI). These regions contain critical motifs in multiple polymerase regions and critical residues involved in capping and methyltransferase activities. A total of 17 codons were predicted to be under positive selection at this gene (Figure 6E). Out of these, eight codons were located at the RNA polymerase domain (RdRp) at codon positions 122, 126, 174, 212, 266, 395, 644, and 695. These codons impact isolates from groups 1, 3, and 4 (Figure S3, Appendix A). Interestingly, mutations at some of these codons (266, 395, 644, and 695) disrupted residues in conserved regions I (CRI) and III (CRIII) (Figure 6E). In this sense, three codons (1251, 1272, and 1298) located at the capping domain (Cap) produced changes at residues of the conserved region V (CRV) (Figure 6E). These mutations were associated with individual isolates of groups 1 and 2 (Figure S4, Appendix A). However, mutation at codon 1298 was also present in all VSIV isolates from Central and South American clades (Figure 1 and Figure S5, Appendix A). Notably, FEL analysis conducted on internal branches detected codon 1298 to be under positive selection (*p*-value = 0.07), indicating the potential of this codon site to induce adaptation at the population level on VSIV in nature.

Three codons (1345, 1590, and 1591) were predicted in linker regions under positive selection (Figure 6E). At codon 1345, mutations were found in two independent isolates from genetic group 1, while single isolates from groups 2 and 3 carried mutations at codons 1590 and 1591 (Figure S4, Appendix A). Finally, the last four codons under positive selection were distributed between methyltransferase (MT) and C-terminal (CTD) domains (Figure 6D). The MT domain included codons 1605 and 1887, where three independent isolates from group 4 and one single isolate were identified as carrying mutations at these codons, respectively (Figure S4, Appendix A). Remarkably, in this domain, the codon L-1784 was predicted under positive selection (Figure 5). The L-1784 codon not only differentiates the endemic ancestral viruses (IN0817CPB and IN1017CPB) from the epidemic lineage by the mutation CGG**_(R)_**−CAG**_(Q)_** but also from the other VSIV previously reported in nature (Figure S4, Appendix A). This codon is part of the conserved region VI (CRVI) (Figure 6E), where CGG**_(R)_** was the dominant allele among VSIV in nature. However, the presence and conservation of allele CAG**_(Q)_** during the circulation of the epidemic lineage in the US stress the potential importance of this mutation in the emergence of this lineage.

The CTD domain included codons 2006 and 2045. Single isolates from groups 3 and 2 were identified with mutations at these codons (Figure S4, Appendix A). Moreover, CTD includes codon 2048 (Figure 6D), identified by our Metadata-driven comparative analysis (Figure 3) and implicated in the divergence within G2 (Figure S2, Appendix A). Despite this codon being classified as a neutrally evolving site by MEME and FEL analyses, mutation at this codon CGT**_(R)_**−CAT**_(H)_** present in all isolates recovered from Kansas and Missouri during the epidemic outbreak in 2020, was also found in the south American isolate 85CLB.

Regarding purifying selection, 30 codons distributed at different functional sites were predicted on the L gene (Figure 6E).

### 3.5. Genetic Distance Within the Epidemic Lineage Positively Correlates with the Geographical Range of Circulation

Finally, another aim of this study was to obtain further insights into potential factors influencing the evolutionary dynamics of this epidemic lineage. Hence, we attempted to determine the correlation between pairwise genetic and geographical distances. We used geographical information associated with the Colorado dataset. This dataset included isolates representing three out of the four main subpopulations identified in this study.

**Figure 7:**
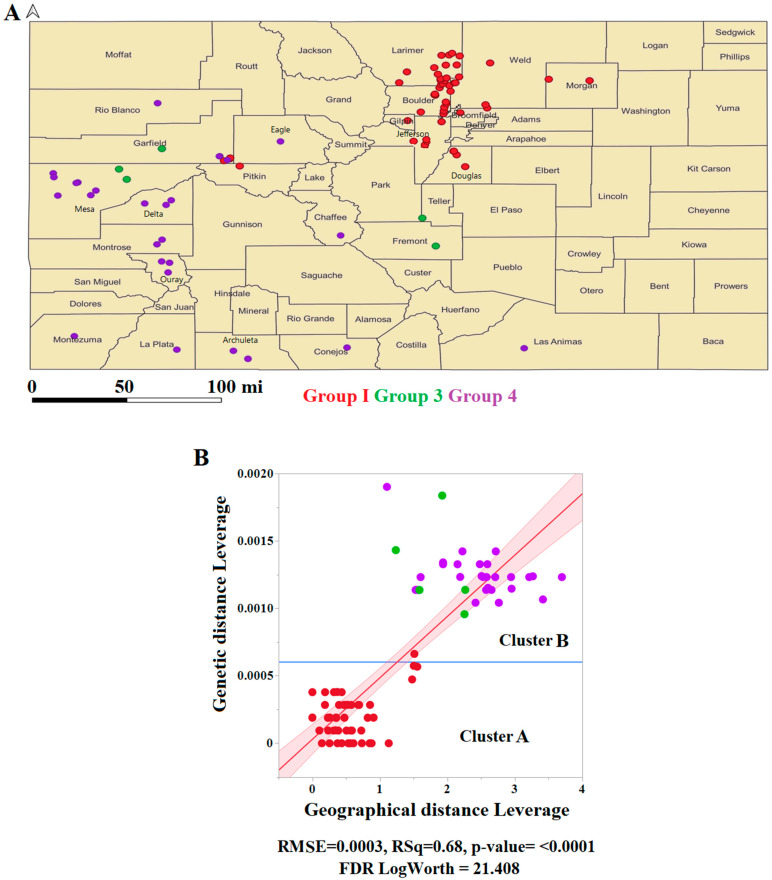
Correlation between genetic and geographical distances using the Colorado dataset as a model. (**A**) Geographical distribution showing counties where isolates belonging to different genetic groups were recovered from naturally infected equine samples in Colorado during 2019. The map was developed using the software QGIS (https://www.qgis.org/en/site/). (**B**) ANOVA analysis was used as an exploratory method to predict the correlation between genetic and geographical distances. RMSE denotes the root mean square error of the model, while RSq indicates the square of the correlation coefficient, and the FDR Log Worth shows the probability that the correlation between variables was caused by chance, with values higher than 2 indicating dependency between variables.

The analysis results indicated that the spatial distribution of groups 1, 3, and 4 was confined to 24 counties, including 45 cities in Colorado (Figure 7A). The linear regression analysis revealed a significantly positive correlation between genetic and geographical distance values (Figure 7B), suggesting that geographical factors play a significant role in the overall genetic variability of this lineage (Figure 7B).

**Figure 8:**
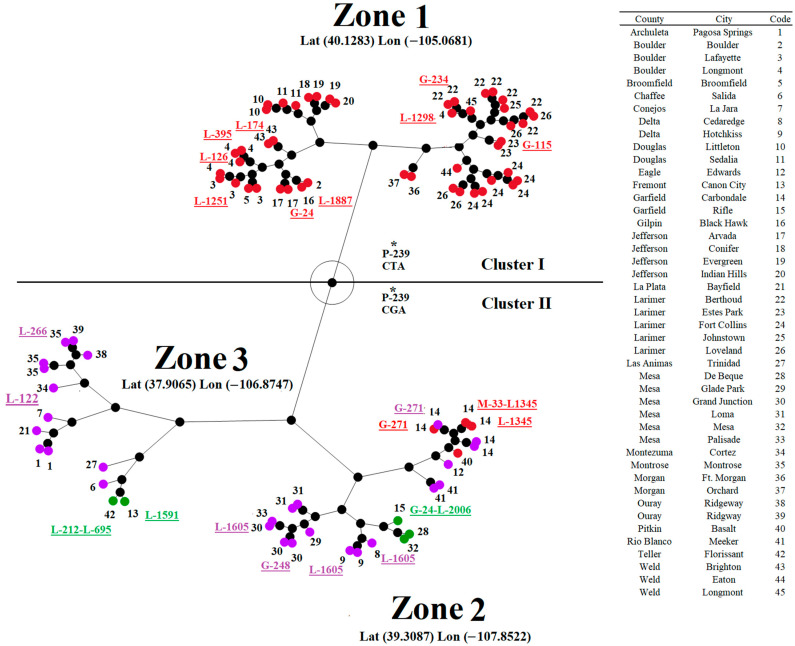
Geographical distribution of isolates displaying codons under positive selection. A constellation plot is shown, depicting the results of a hierarchical cluster analysis based on the latitude and the longitude coordinates where different isolates were collected. Red, green, and purple dots denote isolates belonging to genetic groups 1, 3, and 4, respectively. Different geographical zones determined by ANOVA are indicated. Different codons under positive selection were highlighted next to specific dots to see potential associations between codons at positive selection and their presentation in specific geographical zones. The numbers next to the dots correspond to specific counties and cities in Colorado.

Once we established a positive correlation between genetic and geographical distances, we attempted to understand if positive selection at multiple isolates might be associated with their circulation in specific geographical settings. We conducted a hierarchical cluster analysis (results were presented as a constellation plot) (Figure 8). Subsequently, based on ANOVA and supported by Tukey’s honest significance test (p-0.05), we determined three significant distinct geographical zones of circulation in Colorado (Figure 8). In this context, zone 1 comprised counties and cities associated exclusively with most of the isolates related to the genetic group 1, while zones 2 and 3 included indistinctly isolates from groups 3 and 4. Only four isolates (IN1019COE1, IN10COE5, IN1019COE6, and IN1019COE8) from group 1 circulated in geographical zone 2. Considering the general geographical pattern distribution of different isolates, we may emphasize the potential role of codon P-239 in the adaptation of isolates associated with the two main phylogenetic clusters to circulate in distinct geographical settings (Figure 8).

All codons detected at leaf nodes under positive selection were linked to isolates indistinctly circulating across all three geographical zones. The only exceptions were codons G-24 and G-271. Interestingly, mutations at codon G-24 were associated with isolates from groups 1 (IN0719COE52) and 3 (IN1019COE2), circulating in two distinct geographical zones (1 and 2). Conversely, codon G-271 was associated with two isolates circulating in geographical zone 2 (Figure 8). Interestingly, as previously stated, the selection of this codon involved single isolates from genetic groups 1 and 4. The close geographical proximity between the places where these isolates were circulating may be possible to suggest the potential adaptive role of G-271.

Finally, our analysis revealed a clear association between isolates selecting codons L-1345 and L-1605 and their circulation at specific geographical settings in geographical zone 2. In the case of codon L-1345, the selection of this codon was associated with the circulation of two isolates (IN1019COE5 and IN1019COE6) from group 1, which were circulating at the county of Garfield and the city of Carbondale (Figure 8). Interestingly, the remaining isolate from group I (IN1019COE1) circulating in the geographical zone 2 at the county of Pitkin and the city of Basalt did not show evidence of selection at codon L-1345. The latter suggests that ecological conditions in counties Garfield and Pitkin may represent two different challenges in terms of adaptation.

Nevertheless, the selection of codon L-1605 was also associated with isolates from genetic group 4 circulating in the contiguous counties of Delta and Mesa (geographical zone 2), indicating the potential role of codon L-1605 in the adaptation of these isolates.

## 4. Discussion

The emergence of VSV in the US has been a recurrent event during recent decades [4,6,7,8]. There is solid evidence of the relationship between genetic lineages circulating in Mexico and the ones producing epidemic events in the US [4,6,8]. However, very little is currently known about the role of natural selection in driving the emergence and maintenance of epidemic lineages in the US. Here, based on a representative number of full-length viral sequences, we present for the first time the evolutionary dynamic signatures of an epidemic VSIV lineage that affected the US during 2019–2020.

Consistently with previous studies [4,6,8], our phylogenetic analysis revealed that the closest genetic relative of this epidemic lineage was VSIV isolates circulating in Chiapas—an endemic zone of Mexico. A notable feature of this epidemic lineage is the presence of a nucleotide insertion in the intergenic region between G and L genes, a signature also found in the ancestral isolates from Mexico. This is an interesting finding as insertions in this intergenic region were previously considered a hallmark just for VSIV isolates from a Central America geographical origin [74], indicating that the presence of insertions in intergenic regions is a condition also present in lineages from North America. We currently don’t have an explanation for the possible role of this insertion in the epidemic lineage. Our study identified two genotypes distinguished by a 14-nucleotide and a 22-nucleotide insertion. The presence of these insertions and their apparent stability in the epidemic 2019–2020 lineage during the outbreak, indicate that insertions may be used as potential diagnostic and epidemiologic markers during future VSIV epizootics in the US.

Interestingly, all viruses carrying the 22-nucleotide insertion were categorized into genetic group 3, the less prevalent group during the outbreak. This observation might suggest that the length of insertion may provide a potential adaptive advantage in nature. In vitro, studies have reported the presence of nucleotide insertions in noncoding regions in VSIV genomes in response to evolution on a regimen of constant alternating passages between mammalian and insect cells under laboratory conditions [75], a situation that may be consistent with the evolutionary history an epidemic VSIV lineage in the field.

Although the high degree of conservation of the polyadenylation signal at different intergenic regions in VSV has been reported [41], we found that the length of this signal is not conserved due to the insertion of extra uracil residues in multiple lineages. Interestingly, studies have shown that an increase in the length of the polyadenylation signal from 7 to 14 uracil impaired the ability of the polymerase to initiate downstream mRNA synthesis [41]. Based on this result, we can hypothesize that the observed increase in the uracil tail in a minority of lineages (having 8 and 9 uracil tails) during the outbreak in different intergenic regions might have resulted in a selective disadvantage.

Previous studies have described complex evolutionary dynamics associated with the emergence of arboviral populations, including bottlenecks, founder effects, genetic drift, and natural selection [76,77] (Appendix B). Our results indicated that the epidemic VSIV lineage 2019–2020 diversified into at least four subpopulations during the outbreak. This situation is consistent with previous VSV outbreaks in the US [4,6,8].

We used two different strategies to elucidate the possible role of evolutionary forces in the epidemic dynamics of VSIV lineage 2019–2020. First, we used the Metadata-driven Comparative analysis tool to identify positions in the genome that were significantly different between the four subpopulations. One of the main conclusions obtained from this analysis was the preponderance of a specific group of synonymous mutations in shaping the phylodynamic of the epidemic lineage. Overall, synonymous mutations are often considered neutral regarding adaptation, as they do not alter the encoded amino acid. However, currently, there is substantial evidence suggesting that synonymous mutations can have adaptive effects that enhance the fitness of viral populations under experimental conditions [78]. For instance, research on the influenza virus has shown that its evolution in the presence or absence of antiviral drugs highlights the significance of synonymous mutations, with fitness effects ranging from 5% to 30% [78,79]. Additionally, in vivo experiments involving the introduction of numerous synonymous mutations in specific genomic regions of highly virulent strains of classical swine fever virus [80] and foot-and-mouth disease virus [81] have resulted in attenuated phenotypes in pigs, the natural hosts of these viruses. This indicates that synonymous mutations can influence the virulence of viral populations.

Interestingly, our findings align with a previous study on VSV, which demonstrated a positive selection of synonymous mutations during in vitro passaging experiments conducted in selective environments, such as insect cells or through alternating between mammalian and insect cells [82]. Notably, these synonymous mutations emerged in VSIV populations as a result of parallel evolution, enhancing the fitness of these populations for growth in insect cells [82]. This indicates the potential adaptive role of synonymous mutations in the evolution of the epidemic VSIV lineage in the U.S.

The analysis of ancestral reconstruction revealed that the fixation of these mutations occurred at internal nodes and was preserved after multiple cycles of transmission in the field. This pattern suggests that natural selection, rather than genetic drift, played a significant role. Notably, three synonymous mutations at codons G-439, G-455, and L-689 were found fixed deep within the internal nodes related to the divergence of this epidemic lineage across two main phylogenetic clades. This indicates that these mutations may have been important for the early adaptation of the epidemic lineage in the U.S. Furthermore, some of these synonymous mutations emerged as distinct markers for specific genetic groups. This suggests that synonymous mutations could serve as epidemiologic markers to help track the spread of epidemic VSV lineages in the United States.

Furthermore, the fact that some of these synonymous mutations located at codons P-13, G-381, L-574, L-1913, and L-2021 were found in previous VSIV lineages circulating in Central and South America, strongly suggests that they were the resultant of parallel/convergent evolution, emphasizing their potential relevance in the adaptation of VSIV in nature. However, we currently lack a clear explanation for the possible functional role of synonymous mutations during the evolution of this epidemic lineage. A previous study on VSIV proposed an interesting hypothesis regarding the potential role of synonymous mutations in immune response evasion in insects produced by RNA interference (RNAi) [82]. Additionally, the role of synonymous mutations in escaping host antiviral RNAi immunity has been experimentally probed during infections with the white spot syndrome virus in shrimp [83]. Considering the different number of vectors identified during the VSIV outbreak in the US in 2020 [84], it is feasible to propose the use of the synonymous mutations identified in our study as markers for future studies to test the role of these mutations in evasion of the immune response by VSV in relevant vector species in the field.

Our second strategy focused on using evolutionary methods that detect sites under positive or purifying selection using dN/dS ratios in a codon-based phylogenetic framework. According to these approaches, we can emphasize the role that purifying selection has in the evolution of VSIV in nature. This result is consistent with previous publications indicating the dominance of purifying selection during the evolution of arboviral populations, a condition that has been associated with the need to replicate in both vertebrate and invertebrate hosts [76,85], or to avoid the high rates of deleterious mutations seen during the evolution of arboviruses [86]. Our analysis revealed that the action of purifying selection was not uniform among different viral genes, strongly suggesting that the functionality of its proteins influences the evolutionary dynamics of VSIV in nature. In this context, the strongest levels of purifying selection were inferred in N and M genes, possibly associated with the critical roles of N and M proteins during the infectious cycle of VSV. The N protein plays a crucial role in protecting the viral genome, in addition to transcription and replication activities [87], while the M protein has a significant function in immune evasion [88], viral assembly, and budding [61].

Conversely, we identified P and G genes as the main drivers of the divergence of VSIV in nature. The P protein primarily functions as a polymerase cofactor (linking L–N proteins) for transcription and replication activities and as a chaperone for the proper encapsulation between nascent N proteins and viral RNA [57]. In our study, we identified codon sites linked to specific functional domains where nonsynonymous mutations accumulated during the evolution of the epidemic lineage. Most nonsynonymous mutations were detected in the dimerization domain. In this sense, previous studies showed controversial results about the potential role of this region in viral growth in cell culture [57,89]. However, none of these mutations were subject to positive selection, suggesting the potentially neutral nature of these changes. Previous amino acid comparisons among different vesiculoviruses indicate that nonsynonymous changes found in the epidemic lineage at codon positions 112, 140, 158, and 161 were associated with variable positions at this functional region [57], supporting our hypothesis about the possible neutral effect of these mutations.

On the other hand, the codons under positive selection at P-194 and P-212 were associated with the hinge and domine II regions of the P protein. Interestingly, experimental deletions in these regions involving codons P-194 and P-212 have adverse effects on the replication of VSIV [56], suggesting a potential phenotypic effect of these mutations at these codons. Additionally, our analyses identified under positive selection codon P-239, located in domain II, associated with the P–N binding region. The interaction between P and N proteins in this region plays an important role in the replication of VSIV [59]. Future studies are needed to identify the possible relevance of P-239 in the infectious cycle of VSIV.

The G protein plays a role in receptor recognition on the host cell surface and triggers membrane fusion after endocytosis of the virion [68]. Our findings indicate that the epidemic lineage accumulated multiple codons under positive selection along different functional domains during the outbreak. Among the codons under positive selection, we can highlight codons G-115 and G-271, both associated with epitopes identified during the evolution of VSIV in the presence of polyclonal antibodies [50]. This result suggests that viruses with mutations at these positions may represent neutralization-escaping mutant phenotypes. Furthermore, another variable codon, G-352, was identified in association with an epitope during the evolution of the epidemic lineage [65]. As mentioned above, mutation GCT**_(A)_**−GTT**_(V)_** at codon G-352 was linked to the emergence of subpopulation G2, which was dominant during the 2020 outbreak, suggesting that G2 might have represented a lineage capable of escaping neutralization. However, based on the results of MEME and FEL analyses, which categorized G-352 as a neutrally evolving site, we may suggest that fixation of mutation GCT**_(A)_**−GTT**_(V)_** in G2 might have been a result of genetic drift or founder effects rather than natural selection. This situation is expected during the evolution of arboviral populations [76,77]. The relevance of these results will need to be confirmed through future experimental studies.

The L gene harbored most of the sites identified as being under positive selection during the evolution of this epidemic lineage. Similar to the G gene, mutations were found at multiple functional sites. However, the potential effect of various sites under positive selection found in this study will require future studies to confirm their possible phenotypic effect. Numerous codon sites under positive selection are located in conserved regions of the polymerase CRI, CRIII, CRV, and CRVI. A notable finding in our study was the detection of codon L-1784 at CRVI under positive selection, which may be linked to the emergence of the epidemic lineage. The mutation at codon L-1784 is not associated with any critical residue at CRVI, which functions as [ribose-2′-O]-methyltransferase in the VSV polymerase [71]. L-1784 is close to the highly conserved residue K-1795. Mutations at K-1796 have been associated with defects in plaque formation, replication, and mRNA synthesis [71].

Based on ancestral reconstruction analyses, we were able to describe two phylogenetic patterns during the evolution of the epidemic lineage in the US. The most common pattern involving sites under positive selection was associated with the selection of specific codon sites at the leaf nodes of the phylogenetic tree, denoting the potential adaptation of multiple strains at an individual level. However, considering the lack of persistence of these mutations in the population and based on previous publications [90,91], these mutations might have constituted potential evolutionary dead ends, becoming deleterious in the long term. Alternatively, we observed potential advantageous mutations that recently emerged under outbreak conditions. Many of those mutations are expected to fall first toward leaf nodes rather than internal nodes [90]. An excellent example is the codon under positive selection L-1298, which was tracked at leaf nodes in the epizootic lineage but fixed at internal nodes in viruses of Central and South American origin. It was the only codon identified by FEL under positive selection at internal nodes, supporting the potential relevance of L-1298 in producing adaptation at the population level. Additionally, we identified two codons under positive selection (G-24 and G-271) at the leaf nodes, each associated with two isolates linked to two specific genetic groups, showing the potential adaptive advantage of these mutations. However, due to the limited number of sequences used in our study, these mutations’ true prevalence and recurrence may have been underestimated.

The second pattern was associated with an evolutionary scenario previously described in HIV-1 [91], where from the total of codons identified under positive selection (in our study: P-239, L-1354, and L-1605), only a minimal number of mutations at these codons can be expected to be fixed at internal nodes and persist in the population after multiple transmission cycles, suggesting their potential adaptive advantage at the population level. In our study, we identified some nonsynonymous mutations that, despite being tracked at internal nodes and persisting during multiple transmission cycles, no evidence of positive selection was detected by MEME and FEL analyses in the codons associated with these mutations. This was the case for codons P-112, P-161, and G-352, all detected by our Metadata-driven comparative analysis. We can offer two possible explanations. First, mutations at P-112, P-161, and G-352 might have arisen in the population due to genetic drift or founder effects [77]. Second, dN/dS approaches may lack power for short evolutionary timescales and cannot detect all types of positive selection [91]. For example, codon G-352, which encodes an amino acid linked with a neutralizing epitope [65], has a plausible mechanism for directional selection (escape). However, as mentioned above, since all this methodology is based on in silico approaches, experimental evidence is needed to understand the potential phenotypic effect of mutations at these codons. Additionally, the fact that some mutations including synonymous and nonsynonymous showed a potential ability to produce adaptation at the population level during this VSIV epizootic outbreak has implications for the conduction of future molecular epidemiology analyses associated with the emergence of VSV in the US.

Finally, we found a correlation between geographic and genetic distances among subpopulations that circulated in Colorado. This finding was consistent with a previous study [92], suggesting that ecological factors, rather than temporal ones, play a dominant role in the evolution of VSV in the field. Our analysis supports the potential relevance of some codon sites under positive selection with effects at a population level, like P-239, clearly associated with the circulation of genetic groups G1 and G3–G4 in distant locations. Other important codons, L-1345 and L-1605, potentially favor the adaptation within groups 1 and 4, respectively. Considering that all viral lineages from Colorado were recovered from horses, it is possible to hypothesize that the possible adaptation produced by P-239, L-1345, and L-1605 might have been linked to the need for VSIV to replicate in distinct insect vector populations. This hypothesis finds support in previous studies that reported the presence of VSIV in multiple insect vectors during the epizootic outbreak in 2020 [84,93]. However, it is important to note that the identification of the different geographical zones in Colorado is based solely on significant differences in geographical distances among them; thus, further studies are also needed to better characterize their ecological differences. Future in vivo studies are needed to comprehend the relevance of these codon positions in the adaptation of VSIV to replicate in distinct insect vectors.

Conversely, several codon sites under positive selection associated with individual adaptation appeared randomly distributed along different geographic zones. This suggests that these sites played a limited role in the adaptation of these lineages during the outbreak. However, as mentioned earlier, it is crucial to consider that these mutations’ true prevalence and recurrence may have been underestimated.

## 5. Conclusions

In summary, our study revealed the complex dynamics involving the evolution of a highly conserved epidemic lineage of VSIV in the US. It highlights the importance of natural selection in the adaptation of VSIV in the field, suggesting that a minimal number of changes in the P, G, and L genes at specific functional regions might be responsible for the adaptation of this lineage during the outbreak. Our study indicates the potential significance of the synonymous mutations during the circulation of this lineage in the US. Additionally, we emphasize the importance of using a combined methodology based on dN/dS and detecting mutations that are increasing their frequencies in the population to identify potential key codon positions that are driving the evolution of VSV lineages during epizootic outbreaks. It is important to note that, due to the lack of experimental evidence supporting the significance of the key codon positions identified in this study, further research is needed to evaluate their potential impact on the evolution of VSIV. We consider that the methodology used in this study may serve as a framework for conducting future evolutionary studies on VSV in nature.

## Figures and Tables

**Table 1:** Overview of evolutionary pressures across the genome of VSIV. A dN/dS at each gene was computed using the MG94xREV model. The confidence interval was estimated using profile likelihood.

Gene	Codons	Tree length	dS	dN	dN/dS (95% CI)	Sites Under Selection (*p* ≤ 0.05)		Sites Under Selection/1000 Codons	
						Purifying	Positive	Purifying	Positive
N	422	0.176	0.814	0.011	0.019 (0.01–0.03)	60	1	142.2	2.4
P	265	0.259	0.882	0.092	0.167 (0.13–0.21)	37	4	139.6	15.1
M	229	0.238	1.062	0.032	0.041 (0.03–0.06)	45	2	196.5	8.7
G	511	0.263	0.933	0.089	0.136 (0.11–0.16)	81	11	158.5	21.5
L	2109	0.229	0.921	0.048	0.075 (0.07–0.09)	342	24	162.2	11.4
Genome	3536	0.230	0.912	0.051	0.081 (0.08–0.09)	565	42	159.8	11.9

## Data Availability

All sequences used for this study are available at the GenBank database. The alignments of each gene used to conduct the evolutionary analyses in this study are available at https://github.com/spond/pubs/tree/master/VSIV (accessed on 6 October 2024).

## Supplementary Materials

The following supporting information can be downloaded at: https://www.mdpi.com/article/10.3390/v16111803/s1, Figure S1: Model substitution analysis; Figure S2: Ancestral reconstruction analysis of the SNPs identified by Metadata-driven comparative analysis; Figure S3: Codons under purifying selection predicted by FEL analysis, Figure S4: Ancestral reconstruction analysis of mutations at codons under positive selection identified by MEME analysis; Figure S5: Ancestral reconstruction analysis at codons L-1298 and L-1784.

## Appendix A

Figure S1: The figure shows the results of the comparison among 24 different nucleotide substitution models (conducted on Mega), from which GTR + G was selected as the best selection model for the phylogenetic and ancestral reconstruction analyses presented in this study. Models with the lowest BIC scores (Bayesian Information Criterion) are considered to describe the substitution pattern the best.

Figure S2: The figure shows the ancestral reconstruction analysis conducted at different SNPs identified by the Metadata-driven comparative analysis, showing the ancestral state at different internal and leaf nodes of each genetic group (G1 to G4). Next to each tree (left side) is the information regarding the specific gene-codon where this SNP was identified, nucleotide position at that specific gene, codon change (capital letter represents the position where SNPs were found at specific codons), and information about this specific mutation other VSIV not related to the VSIV 2019–2020 outbreak in the US. Red asterisks indicate the specific internal nodes in the tree where mutations at specific codons were tracked in the ancestral reconstruction analysis.

Figure S3: The figure shows specific codon sites at N, P, M, G, and L genes predicted under purifying selection by FEL analysis. This analysis was conducted using the alignment available at https://github.com/spond/pubs/tree/master/VSIV (accessed on 6 October 2024). Sites under purifying selection may represent potential functional sites at different proteins, preserved at VSIV during its evolution in nature.

Figure S4: The figure shows the ancestral reconstruction analysis conducted at different sites involving codons identified under positive selection by MEME analysis in the VSIV epidemic lineage. This analysis was conducted using the alignment available at https://github.com/spond/pubs/tree/master/VSIV (accessed on 6 October 2024). Different genetic groups identified in this study G1 to G4 are highlighted in the tree by color branches in red, blue, green, and purpure, respectively. Next to each tree (left side) is the information regarding the specific gene codon which was identified under positive selection. Black asterisks indicate the specific internal or leaf nodes in the tree where mutations at specific codons under positive were tracked in the ancestral reconstruction analysis. Overall, all mutations at codons under positive selection were tracked in the leaf nodes, the only exceptions were codons P-239, L1345, and L-1605, where mutations were also tracked at internal nodes.

Figure S5: The figure shows the ancestral reconstruction analysis conducted on SLAC at codons L-1298 and L-1784. L-1298 was the only codon identified under positive selection at internal nodes by FEL analysis, indicating its potential relevance in the adaptation of VSIV at the population level. During the VSIV outbreak in the US in 2019–2020, we detected L-1298 under positive selection at a single leaf node (IN0719COE12). Interestingly, L-1298 appeared under positive selection in VSIV populations of Central and South America. The selection of codon ACC**_(T)_** was tracked at internal nodes (that represent the sequences of the predicted ancestral VSIV circulating during the outbreak), indicating its potential relevance in the adaptation of VSIV at the population level. On the other hand, the detection of the positive section of codon 1784 should be considered a relevant finding, since mutation CGG **_(R)_**-CAG **_(Q)_** is highly conserved in all viruses associated with the epidemic lineage, stressing its possible relevance in the emergence of the epidemic VSIV lineage in the US.

## Appendix B. Definitions Adapted to This Study

Positive selection or episodic diversifying selection: Natural selective force that promotes adaptation and innovation by increasing amino acid diversity in a viral protein.

Purifying or negative selection: Natural selective force that favors the amino acid conservation in a viral protein. It results in a stabilizing selection that preserves the functionality of a protein by removing deleterious alleles from the viral population.

Genetic drift: Random mutations that occur in the viral genome due to chance or random sampling. Genetic drift reduces the efficiency of selection and genetic diversity.

Population bottlenecks: Represent a form of genetic drift which produces an important reduction in the population size of organisms, resulting in the fixation of mutations at random, and a loss in genetic diversity. Replication in insect vectors represents an important source of population bottlenecks for arboviruses.

Founder effects: A form of genetic drift that results in the fixation of random mutations. It leads to the loss of genetic variability in viral populations. In arboviruses, it may happen when a susceptible host is infected with a specific viral quasipecies derived from a bottleneck after replication in an insect vector. Then, this host moves into a different geographically isolated area, producing the infection of new susceptible hosts, with the consequent dominance of this viral quasipecies in this area. https://veg.github.io/selection-tutorial (accessed on 6 October 2024).
