# Supplementary figures and images for: Phylogenomic Signatures of a Lineage of Vesicular Stomatitis Indiana Virus Circulating During the 2019–2020 Epidemic in the United States
Source: Viruses. 2024 Nov 20;16(11):1803. doi: 10.3390/v16111803 (PMC11598840; doi:10.3390/v16111803)

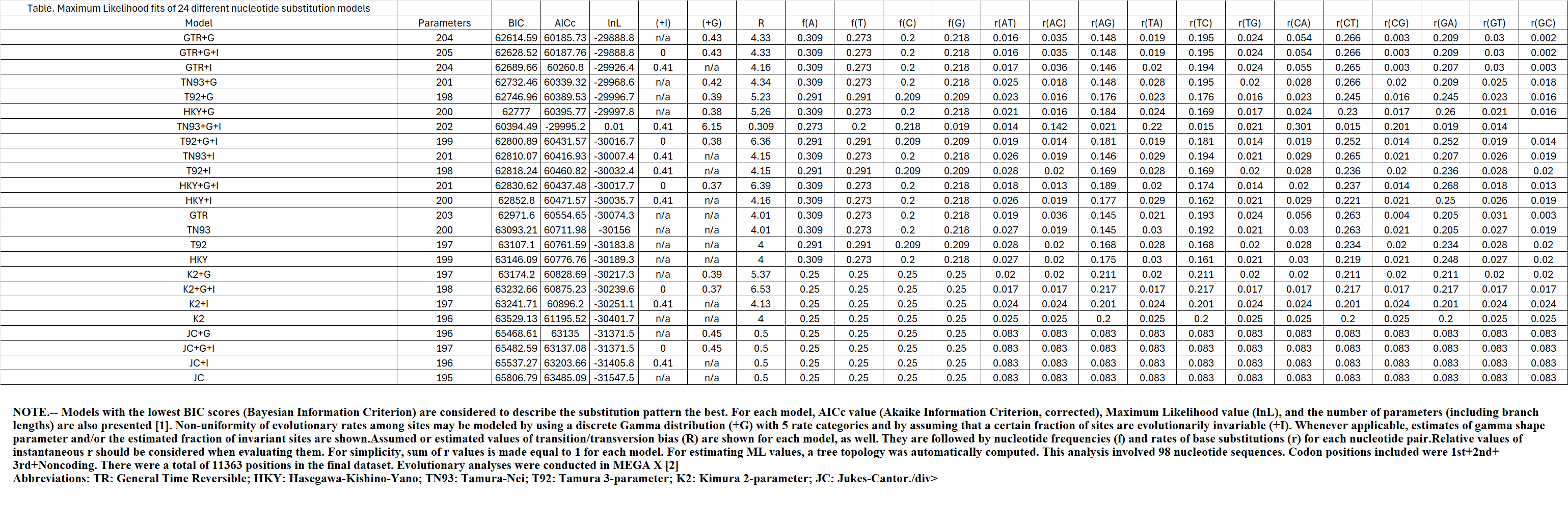

Supplement: Supplementary file 1 [file viruses-16-01803-s001.zip › Figure S1.tif]

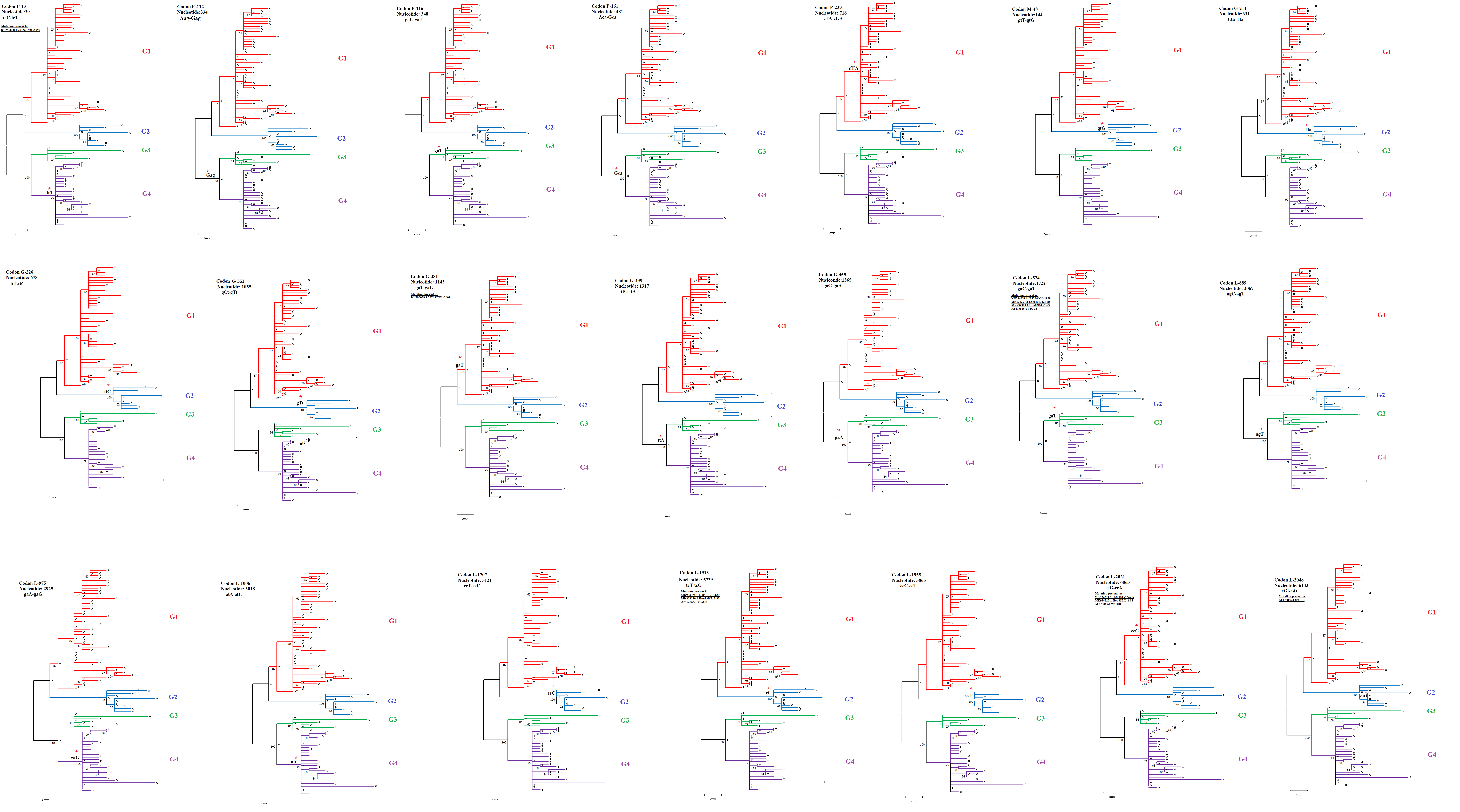

Supplement: Supplementary file 1 [file viruses-16-01803-s001.zip › Figure S2.tif]

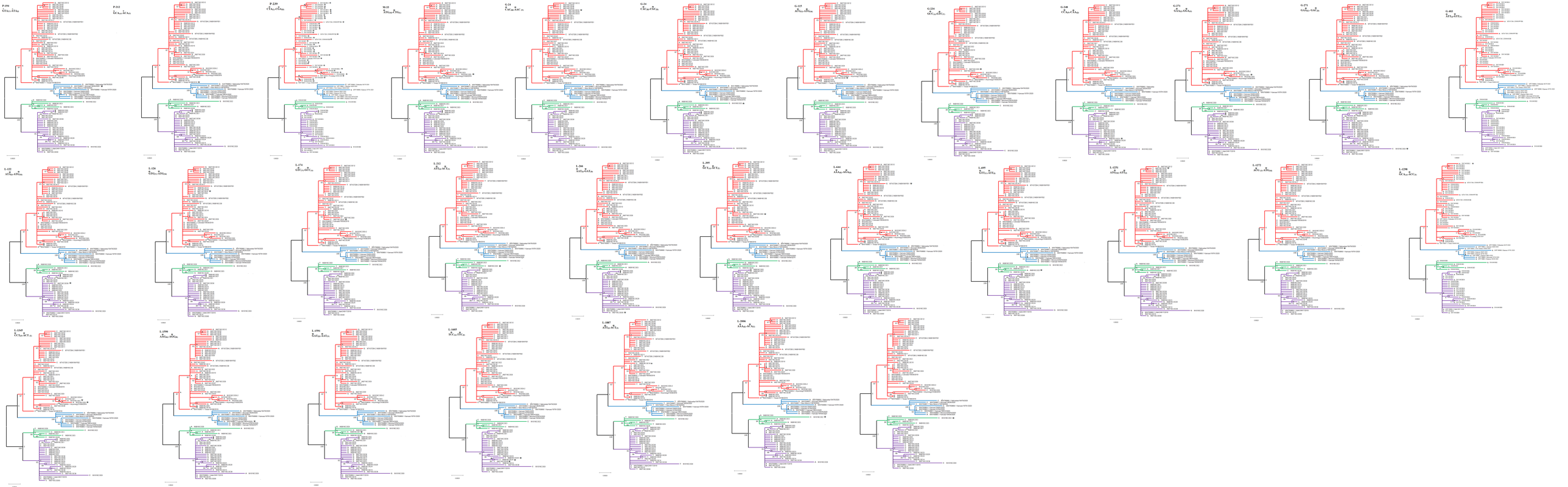

Supplement: Supplementary file 1 [file viruses-16-01803-s001.zip › Figure S4.tif]

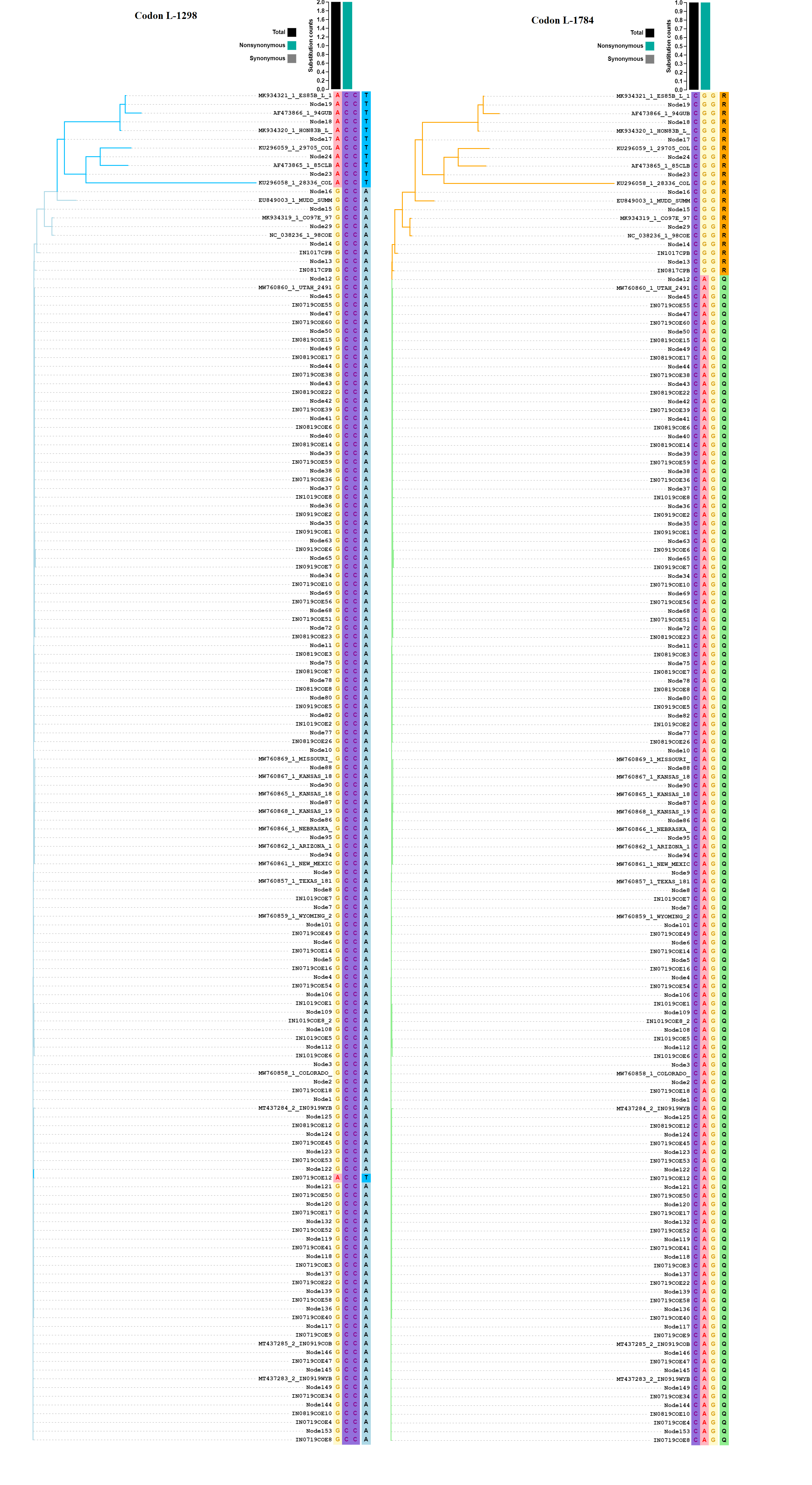

Supplement: Supplementary file 1 [file viruses-16-01803-s001.zip › Figure S5.tif]
